# Artificial intelligence-assisted endoscopic ultrasound diagnosis of esophageal subepithelial lesions

**DOI:** 10.1007/s00464-025-11767-5

**Published:** 2025-05-07

**Authors:** Ai-meng Zhang, Dai-min Jiang, Shu-peng Wang, Wen Liu, Bei-bei Sun, Zhe Wang, Guo-yi Zhou, Yao-fu Wu, Qing-yun Cai, Jin-tao Guo, Si-yu Sun

**Affiliations:** 1 Department of Gastroenterology, Shengjing Hospital of China Medical University, No. 36, Sanhao Street, Shenyang, 110004 Liaoning Province China; 2 Research Center for Innovation, SonoScape Medical Corporation, Shenzhen, 518107 Guangdong Province China; 3 Department of Pathology, Shengjing Hospital of China Medical University, Shenyang, 110004 Liaoning Province China; 4 Digital Information Development Department, SonoScape Medical Corporation, Shenzhen, 518107 Guangdong Province China; 5 Product Management Department, SonoScape Medical Corporation, Shenzhen, 518107 Guangdong Province China; 6 Engineering Research Center of Ministry of Education for Minimally Invasive, Gastrointestinal Endoscopic Techniques, Shenyang, 110004 Liaoning Province China

**Keywords:** Artificial intelligence, Endoscopic ultrasound, Esophagus, Subepithelial lesion

## Abstract

**Background:**

Endoscopic ultrasound (EUS) is one of the most accurate methods for determining the originating layer of subepithelial lesions (SELs). However, the accuracy is greatly influenced by the expertise and proficiency of the endoscopist. In this study, we aimed to develop an artificial intelligence (AI) model to identify the originating layer of SELs in the esophagus and evaluate its efficacy.

**Methods:**

A total of 1445 cases of esophageal SELs were used to develop the model. An AI model stemming from YOLOv8s-seg and MobileNetv2 was developed to detect esophageal lesions and identify the originating layer. Two seniors and two junior endoscopists independently diagnosed the same test set.

**Results:**

The precision, recall, mean average precision @ 0.5, and F1-score of the AI model were 92.2%, 73.6%, 0.832, and 81.9%, respectively. The overall accuracy of the originating layer recognition model was 55.2%. The F1-scores of the second, third, and fourth layers were 47.1%, 51.7%, and 66.1%, respectively. The accuracy of the AI system in differentiating layers 2 and 3 from four was 76.5% and was similar to that of senior endoscopists (74.9–79.8%, *P* = 0.585) but higher than that of junior endoscopists (65.6–66.7%, *P* = 0.045).

**Conclusions:**

The EUS-AI model has shown high diagnostic potential for detecting esophageal SELs and identifying their originating layers. EUS-AI has the potential to enhance the diagnostic ability of junior endoscopists in clinical practice.

**Graphical abstract:**

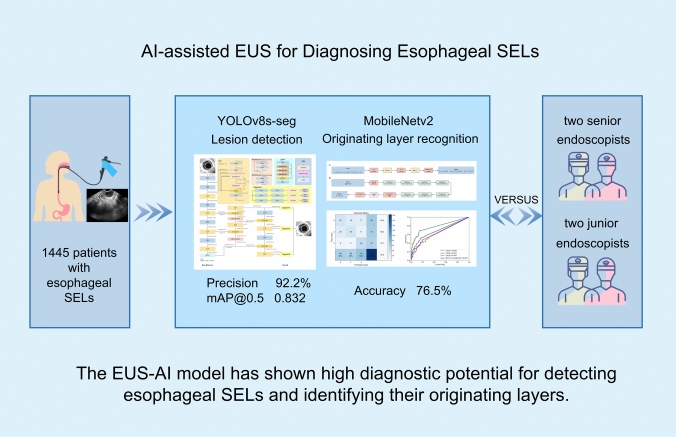

**Supplementary Information:**

The online version contains supplementary material available at 10.1007/s00464-025-11767-5.

Most esophageal subepithelial lesions (SELs) are benign, such as lipoma, leiomyoma, and schwannoma. However, SELs such as gastrointestinal stromal tumors (GISTs) and granular cell tumors can undergo malignant transformation [1, 2]. In current guidelines, there is no consensus on the management of esophageal SELs [1, 3, 4]; however, some suggest that asymptomatic, hypoechoic, well-defined (no high-risk features on endoscopic ultrasound [EUS]) SELs with a diameter of < 2 cm can be managed with continuous surveillance strategy [4]. Nevertheless, extended patient monitoring causes apprehension regarding adverse events, cost-effectiveness, risks of repeat endoscopy, and treatment delay of potentially malignant lesions. Therefore, for the pathological diagnosis and treatment of esophageal SELs, surgical resection remains a crucial approach [5]. There are several techniques for the endoscopic resection of SELs. For example, endoscopic mucosal resection [6, 7] is commonly performed on lesions in the second and third layers. The fourth layer is treated using submucosal tunnel endoscopic resection [8, 9], endoscopic submucosal dissection [10], or endoscopic full-thickness resection [11, 12]. Therefore, the preoperative diagnosis of the originating layer is critical in selecting the best treatment approach [3, 13, 14]. Moreover, grouping the second and third layers versus isolating the fourth layer can accurately match therapeutic decision trees, preventing overtreatment or undertreatment.

Due to the rapid advancement of endoscopic technology and health consciousness among individuals, an increasing number of esophageal SELs have been detected during examination, with an increased demand for EUS diagnosis [15]; however, there is a significant shortage of endo-sonographers. To assess the competency in EUS, the American Society for Gastrointestinal Endoscopy recommends 225 cases [16], whereas the European Society of Gastrointestinal Endoscopy performing at least 250 cases [17]. However, a considerable number of endoscopists have limited practical training, and tertiary referral hospitals house skilled endo-sonographers [18–20]. For amateurs, the learning curve of EUS technology is steep, the diagnostic level varies, and the training system requires improvement [21, 22].

In recent years, artificial intelligence (AI) has made significant advances in medical science [23, 24]. Currently, the role of AI and EUS in diagnosing digestive system disorders has received considerable attention in medical research [25, 26]. Recent research has predominantly focused on GISTs, and the supplementary role of AI in diagnosing esophageal SELs has not been investigated. In this study, we aimed to develop AI models that detect and classify the originating layers of esophageal SELs and evaluate their diagnostic efficacy.

## Materials and methods

### EUS image selection

In this retrospective study, EUS images of esophageal SELs were collected at Shengjing Hospital of China Medical University from January 2015 to September 2023.

Inclusion criteria:

A. Images of esophageal SELs that were either diagnosed and resected (confirmed by pathology) or unanimously diagnosed by two specialist endo-sonographers (with over 10 years of EUS diagnosis experience and more than 5000 EUS procedures performed).
B. Complete EUS examination data.

Exclusion criteria:

A. Poor quality or duplicate images.
B. Images with artificial markers.

This study was approved by the Medical Ethics Committee of Shengjing Hospital of China Medical University (No. 2023PS1355K).

### Image processing

Two specialist endo-sonographers employed Sonokit software (SonoScape Medical Corp., Shenzhen, China) to mark the boundary contour, short and long axes, and originating layer of the esophageal SELs (second layer/deep mucosa, third layer/submucosa, and fourth layer/lamina propria) in each image. Any two identical diagnoses were considered conclusive. However, with different diagnoses, the final diagnosis was referred to an authoritative expert in EUS (over 10 years of EUS diagnosis experience and > 5000 EUS examinations) for further discussion.

### Deep learning algorithms

#### Lesion detection algorithm

**Fig. 1:**
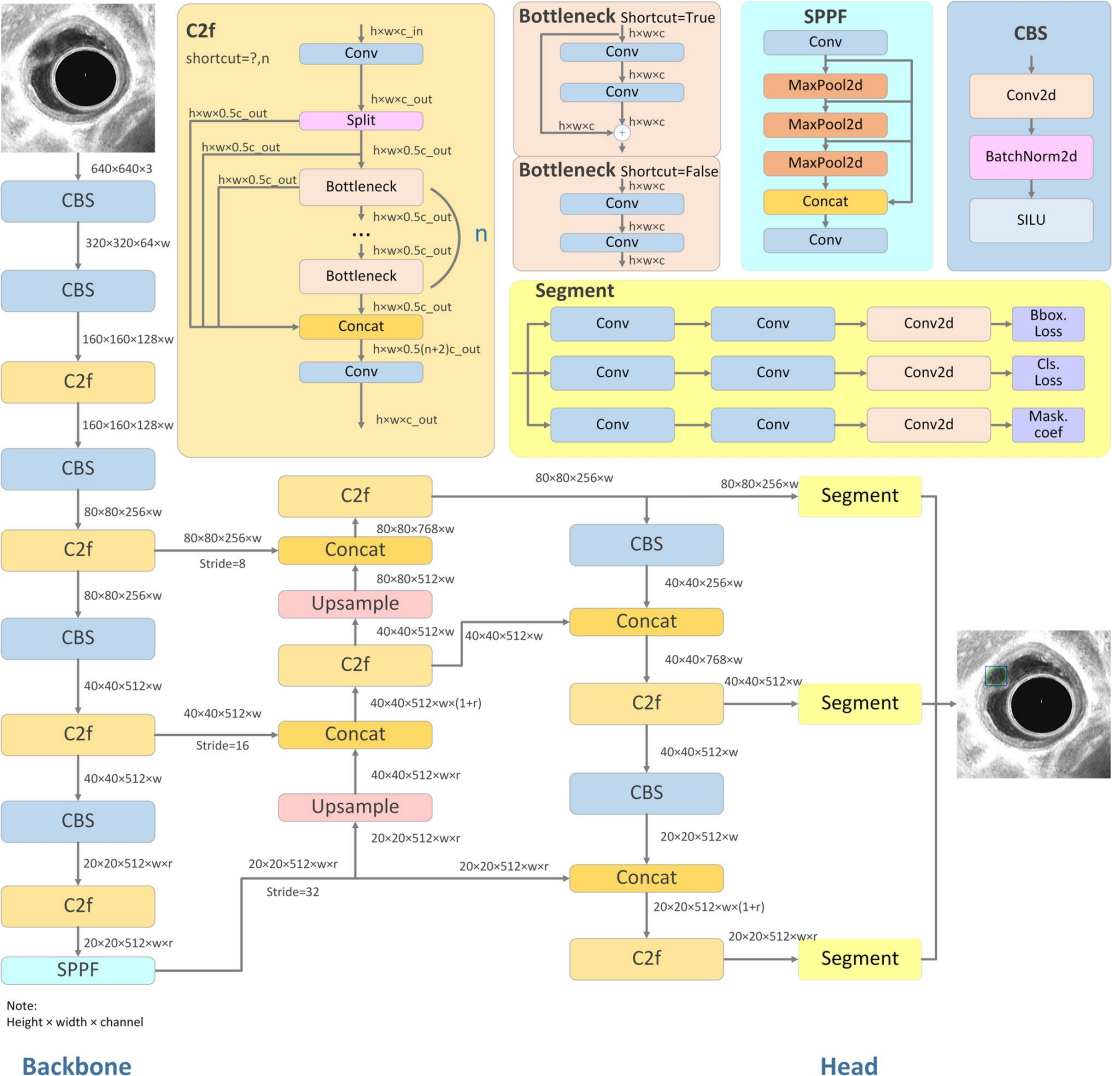
The architecture of YOLOv8s-seg

In order to identify the best AI model, we developed three distinct AI frameworks: YOLOv8s-seg, YOLOv8, and YOLOv5s. The overall performance of YOLOv8s-seg was comparatively superior to the other convolutional neural network (CNN) architectures (Table S1). Consequently, YOLOv8s-seg was selected for the subsequent study. YOLO [27] is a real-time target detection algorithm that utilizes CNN for feature extraction and the identification of species and locations. YOLOv8 builds on the previous YOLO series by adopting its C2f structure as the new backbone network. It introduced a new anchor-free detection header that does not rely on an anchor box. It applies a loss function with a task-aligned assigner-positive sample allocation strategy to introduce the Distribution Focal Loss as a regression loss. YOLOv8s-seg provides better detection and segmentation results while maintaining a fast processing speed. The architecture of YOLOv8s-seg is illustrated in Fig. 1.

#### Originating layer recognition algorithm

**Fig. 2:**
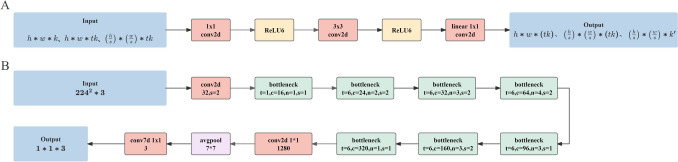
The architecture of MobileNetv2. **A** Bottleneck residual block. **B** Overall network structure of MobileNetv2

In recognition of the originating layers, a classification network called MobileNetv2 performed better than did the other CNNs (Resnet50, EfficientnetB0) (Table S2). It is a lightweight convolutional neural network developed by Google in 2017 and was designed for use on mobile or embedded devices. MobileNetv2’s linear bottleneck layer [28] substitutes the ReLU after a 1 × 1 depth-separable convolution with a linear activation function. A reverse residual structure was employed, which upscaled the 1 × 1 convolution before performing depth-separable convolution. Finally, a Shortcut structure was added to link the 1 × 1 convolution preceding the upscaled 1 × 1 convolution to the 1 × 1 convolution following the 1 × 1 convolution within the depth-separable convolution. The architecture of MobileNetv2 is illustrated in Fig. 2.

### Model construction

The labeled EUS images were randomly divided into training, validation, and test sets in a ratio of 8:1:1 at the patient level, ensuring that there was no intra-patient leakage. Subsequently, we constructed the lesion detection and originating layer recognition models. The training set images were employed to learn the characteristics of the esophageal SELs. The hyperparameters were optimized using the validation set images (Table S3). The test set images helped to assess the diagnostic capability of the optimal model for esophageal SELs.

### Model testing and comparison of diagnostic efficacy with endoscopists

Model testing: The location and originating layer of SELs were diagnosed by AI.

Physician identification: Image boundary contour and originating layer labeling were performed by two senior endoscopists (> 5 years of EUS experience and 500 EUS examinations) and two junior endoscopists (less than 1 year of EUS experience and less than 200 EUS examinations). None of the endoscopists were involved in selecting or labeling the dataset, and the clinical characteristics and the results of endoscopic and pathological examination remained unknown.

### Evaluation metrics

The performance of the AI model was measured with the precision (*P*), recall (*R*), mean average precision (mAP), and F1-score. The F1-score is the harmonic mean of precision and recall. A precision-recall curve (P-R curve) was constructed. The term “Average Precision” (AP) represents the area under the curve. The mean precision of all sample categories produced a weighted average. This metric was employed to assess the detection capabilities across all the categories. In this study, the AP was equivalent to the mAP. The receiver operating characteristic curve (ROC curve) was illustrated, and the area under the curve (AUC) was calculated. The formulas for the related concepts are as follows:

1 $$Accuracy=\frac{TP+TN}{TP+TN+FP+FN}$$

2 $$Precision=\frac{TP}{TP+FP}$$

3 $$Recall=\frac{TP}{TP+FN}$$

4 $$F1=\frac{2TP}{2TP+FP+FN}$$

5 $$AP={\int }_{0}^{1}P(R)dR$$

A true positive (TP) signifies that the genuine category of the sample is positive, and the model predicts positive cases. A true negative (TN) indicates that the genuine category is positive and the prediction result is negative. A false positive (FP) means that the genuine category is negative and the prediction result is positive. A false negative (FN) signifies that the genuine category is negative and the prediction result is negative. Furthermore, the accuracies of the AI model and endo-sonographers in diagnosing esophageal SELs were appraised with 95% confidence intervals (CI). The time required to diagnose the same test set was measured by both groups.

### Statistical methods

Statistical analyses were carried out using SPSS Statistics version 26.0 by the author (A-MZ). Categorical variables were compared using the two-paired McNemar’s test, with statistical significance set at *P* < 0.05. The Kappa test was used to evaluate the consistency of endoscopists’ diagnostic results: *κ* ≥ 0.75, good agreement; 0.75 > *κ* ≥ 0.4, moderate agreement; *κ* < 0.4, poor agreement.

## Results

### General information

**Fig. 3:**
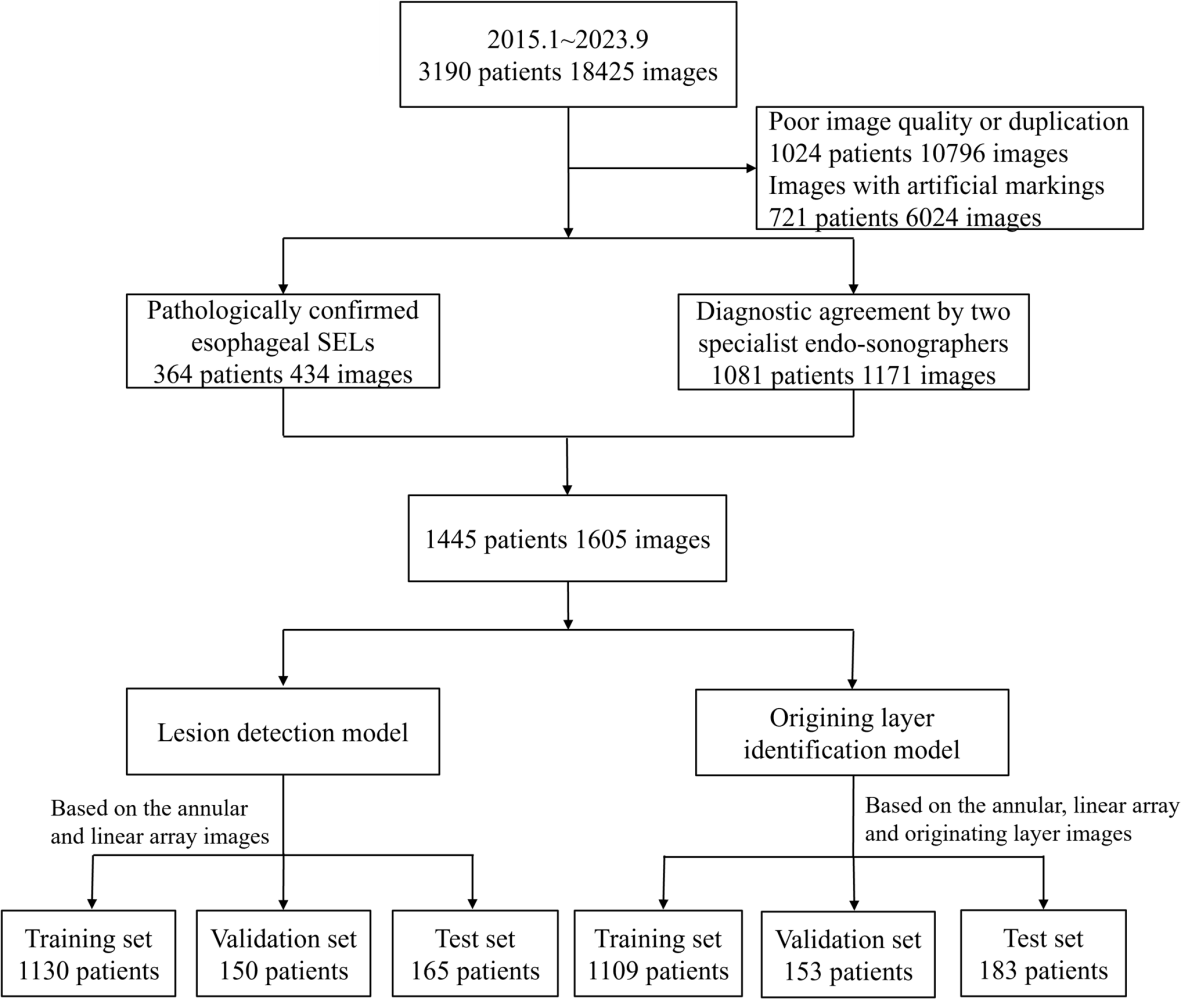
Case inclusion, exclusion, and grouping flowchart

A total of 1445 patients diagnosed with esophageal SELs using EUS were enrolled. In addition, 1605 EUS images were labeled by endoscopists. Table 1 presents the precise distribution of the data. The data were randomly assigned by a computer into training, validation, and test sets (Fig. 3). Table 2 shows the cohort characteristics of patients for each group.

**Table 1:** Number of images for each type of data for esophageal SELs

	The second layer	The third layer	The forth layer
Annular array EUS	526	475	462
Linear array EUS	25	45	72

*SEL* Subepithelial lesion, *EUS* endoscopic ultrasound

**Table 2:** Clinical characteristics of patients for each dataset of AI models

Characteristics	Lesion detection algorithm			Originating layer recognition algorithm		
	Training Set (*n* = 1130)	Validation Set (*n* = 150)	Test Set (*n* = 165)	Training Set (*n* = 1109)	Validation Set (*n* = 153)	Test Set (*n* = 183)
Age, Years (mean ± SD)	54.6 ± 10.8	54.2 ± 11.1	53.3 ± 11.1	54.5 ± 11.1	54.2 ± 9.7	53.9 ± 10.5
Sex						
Male	600 (53.1%)	83 (55.3%)	84 (50.9%)	594 (53.6%)	84 (54.9%)	90 (49.2%)
Female	530 (46.9%)	67 (44.7%)	81 (49.1%)	515 (46.4%)	69 (45.1%)	93 (50.8%)
Tumor size, mm (mean ± SD)	8.3 ± 7.7	9.4 ± 10.9	9.4 ± 8.4	8.5 ± 8.2	7.9 ± 7.7	9.1 ± 8.1
Tumor location						
Upper esophagus	279 (24.7%)	34 (22.7%)	46 (27.9%)	274 (24.7%)	36 (23.5%)	48 (26.2%)
Middle esophagus	278 (24.6%)	52 (34.7%)	60 (36.4%)	372 (33.5%)	54 (35.3%)	62 (33.9%)
Lower esophagus	473 (50.7%)	64 (42.6%)	59 (35.7%)	463 (41.8%)	63 (41.2%)	73 (39.9%)

*AI* artificial intelligence, *SD* standard deviation

### Lesion detection model

**Fig. 4:**
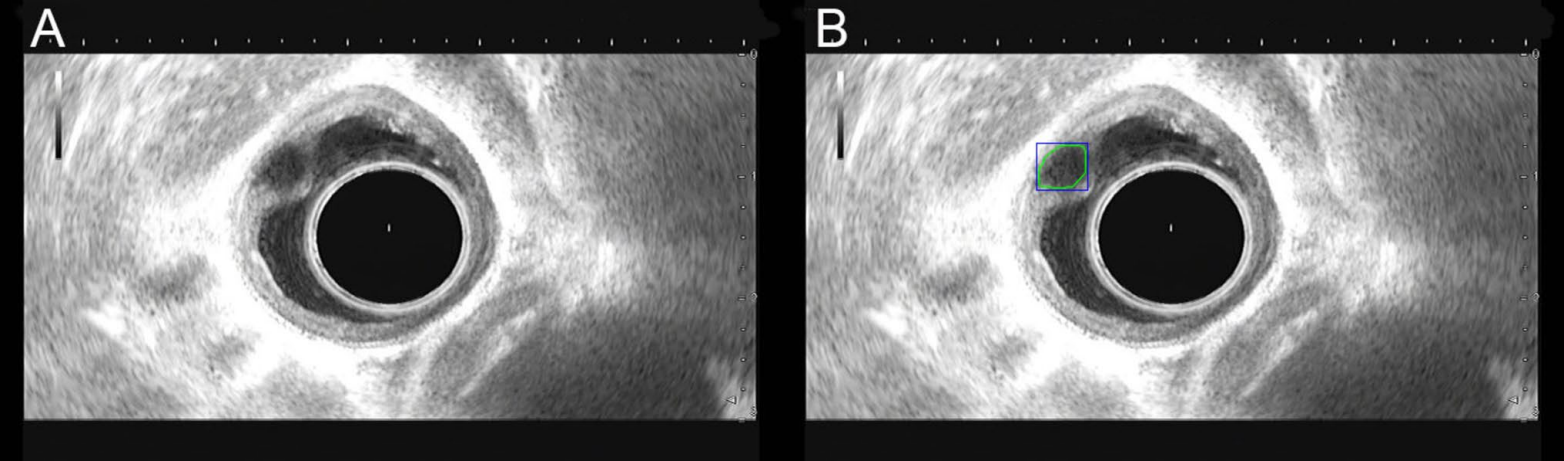
Example of test set image labeling. **A** Representative image of esophageal SELs. **B** AI model recognition image. The green frame indicates the diagnosis by the AI system. *SEL* subepithelial lesion, *EUS* endoscopic ultrasound

The precision of the lesion location detection was 92.2% (95%CI, 87.0–95.3%), the recall was 73.6% (95%CI, 66.1–79.5%), mAP@0.5 was 0.832, and F1-score was 81.9% (95%CI, 75.2–87.0%). Figure 4 shows an example of esophageal SEL recognition using an EUS-AI image model.

The EUS-AI model illustrated an accuracy of 73.6%, which was similar to the senior endoscopists’ range of (71.5–80.6%) (*P* = 0.146). In contrast, the accuracy was significantly greater than that of junior endoscopists (61.8–65.5%, *P* = 0.041) (Table 3).

**Table 3:** Comparison of diagnostic performance between the lesion detection model and endoscopists

Diagnostician	Accuracy, % (95%CI)	*P*^a^
AI model	73.6(66.1–79.5)	Reference
All senior endoscopists	76.1(69.3–81.6)	0.146
Senior endoscopist 1	71.5(64.2–77.9)	0.649
Senior endoscopist 2	80.6(73.9–85.9)	0.245
All junior endoscopists	63.6(56.2–70.0)	0.041^*^
Junior endoscopist 1	65.5(57.9–72.3)	0.086
Junior endoscopist 2	61.8(54.2–68.9)	0.026^*^

*CI* confidence interval, *AI* artificial intelligence

^a^Comparing statistical differences of accuracy between endoscopists and lesion detection models

*P < 0.05

### Originating layer recognition model

#### Ternary-category classification performance

**Fig. 5:**
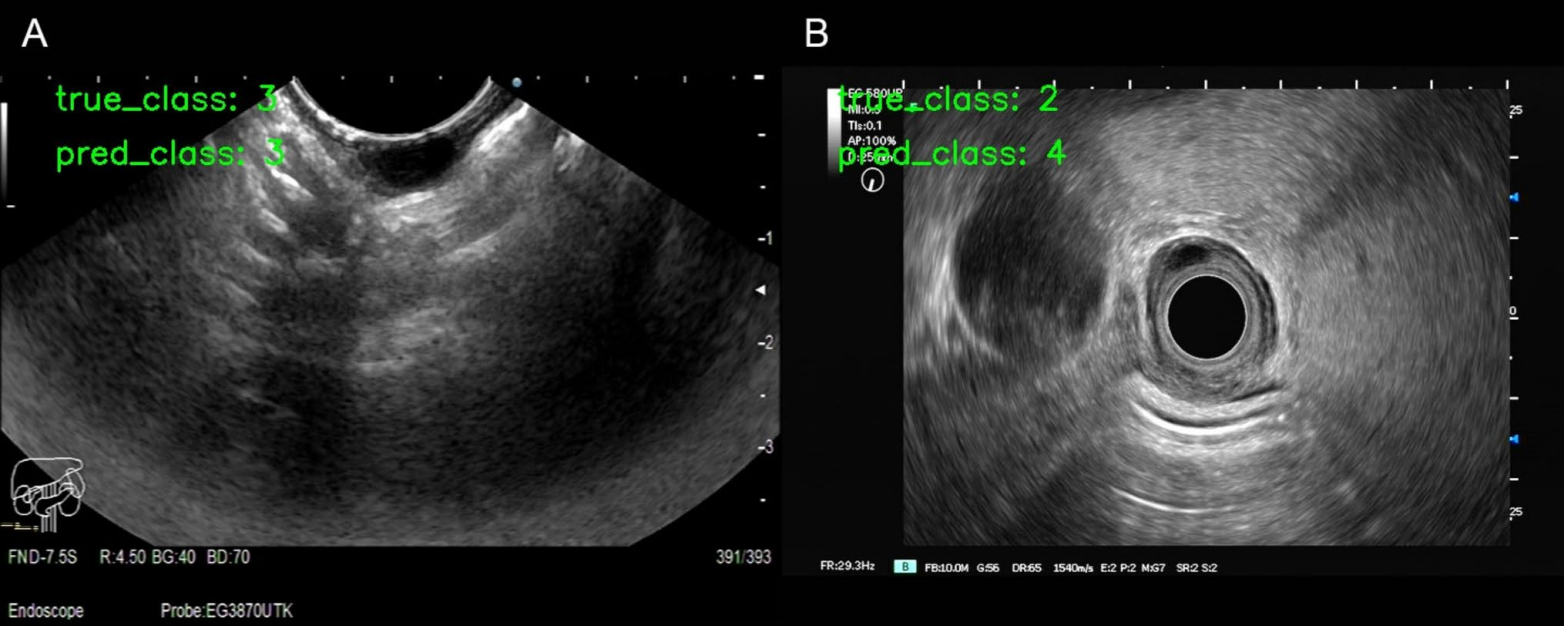
Example of origin-level prediction results. **A** The AI model predicted the third layer lesion as the correct layer. **B** The AI model misdiagnosed the second layer lesion as the fourth

**Fig. 6:**
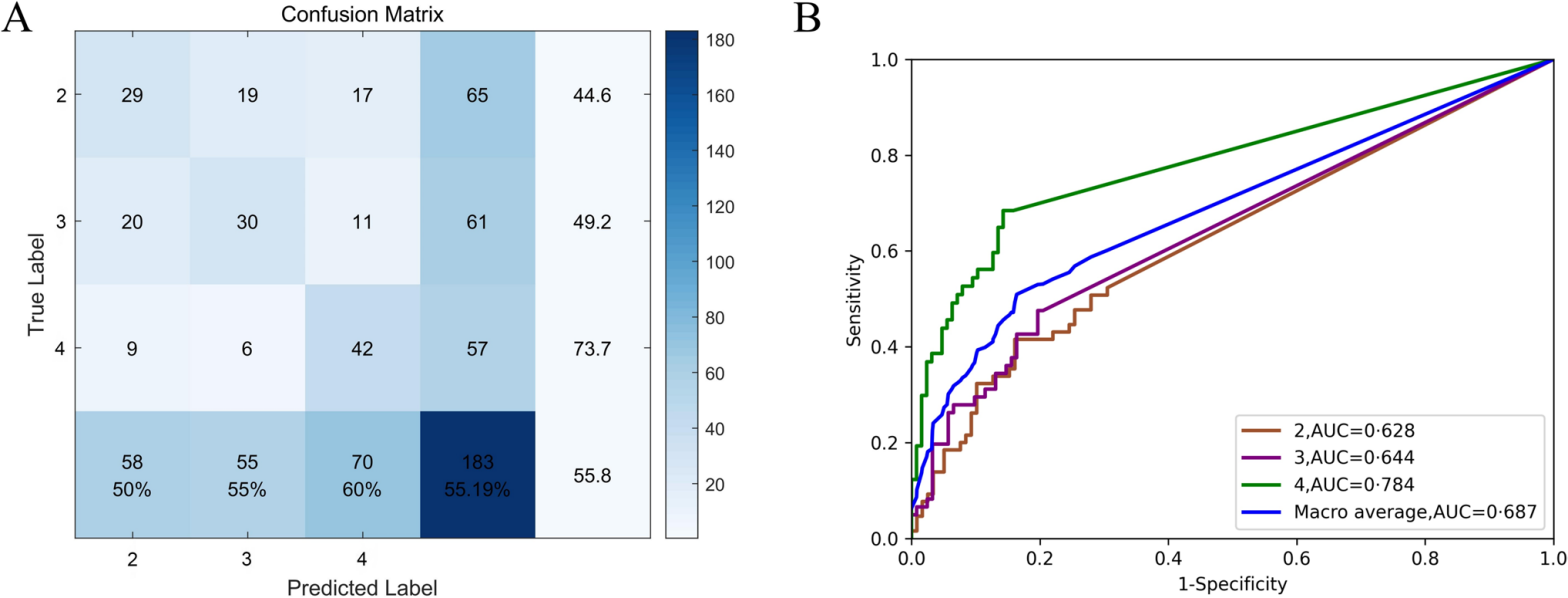
Diagnostic performance of the originating layer recognition model. **A** The confusion matrix of the originating layer recognition model. The originating layers were 2, 3, and 4, with a precision of 50.0, 54.5, and 60.0%, respectively, and a recall of 44.6, 49.2, and 73.7%, respectively. **B** ROC curves of the originating layer recognition model. The AUC for the second, third, and fourth layers was 0.628, 0.644, and 0.784, respectively

The originating layer recognition model’s overall accuracy was 55.2% (95%CI, 48.0–62.2%). Figure 5 shows an example of the prediction results. The originating layers were 2, 3, and 4, with precisions of 50.0% (95%CI, 37.5–62.5%), 54.5% (95%CI, 41.5–67.0%), and 60.0% (95%CI, 48.3–70.7%), respectively; moreover, the recalls were 44.6% (95%CI, 33.2–56.7%), 49.2% (95%CI, 37.1–61.4%), and 73.7% (95%CI, 61.0–83.4%), respectively. The F1-score of the second, third, and fourth layers was 47.1% (95%CI, 39.9–54.2%), 51.7% (95%CI, 44.7–59.0%), and 66.1% (95%CI, 59.0–72.6%), respectively. The confusion matrix is shown in Fig. 6A. The AUC for the ternary-category classification was 0.687 (Fig. 6B).

#### Binary category classification performance

Endoscopic resection of esophageal SELs originating from the muscularis propria (fourth layer) differs from the lesions in the second and third layers. Resection of these lesions can be difficult. Consequently, we categorized the identification of the originating layer into the second or third layer and the fourth layer. The accuracy of the EUS-AI system in distinguishing the fourth layer from the second or third layers was 76.5% (95%CI, 69.9–82.1%) with an AUC of 0.784 (Fig. 5B).

#### Comparison of diagnostic performance between AI model and endoscopists

The accuracy of the AI model (55.2%) in the ternary-category did not show a significant difference when compared with senior endoscopists (53.6–65.0%, *P* = 0.057) and was superior to that of junior endoscopists (42.1–48.1%, *P* = 0.043). In the binary category classification, the accuracy of the AI model (76.5%) was similar to that of the senior endoscopists (74.9–79.8%, *P* = 0.585) but higher than that of the junior endoscopists (65.6–66.7%, *P* = 0.045) (Table 4).

**Table 4:** Comparison of efficacy between the originating layer identification model and endoscopists

	Diagnostician	Accuracy, % (95%CI)	*P*^a^
Ternary	AI model	55.2(48.0–62.2)	Reference
	All senior endoscopists	59.3(51.8–65.9)	0.057
	Senior endoscopist 1	53.6(46.3–60.6)	0.255
	Senior endoscopist 2	65.0(57.9–71.6)	0.058
	All junior endoscopists	45.1(37.8–52.0)	0.043^*^
	Junior endoscopist 1	48.1(41.0–55.3)	< 0.001^***^
	Junior endoscopist 2	42.1(35.2–49.3)	0.098
Binary	AI model	76.5(69.9–82.1)	Reference
	All senior endoscopists	77.4(70.4–82.5)	0.585
	Senior endoscopist 1	74.9(68.1–80.6)	0.306
	Senior endoscopist 2	79.8(73.4–85.0)	> 0.999
	All junior endoscopists	66.2(59.0–72.6)	0.045^*^
	Junior endoscopist 1	66.7(59.6–73.1)	0.047^*^
	Junior endoscopist 2	65.6(58.4–72.1)	0.018^*^

*CI* confidence interval, *AI* artificial intelligence

^a^Statistical differences in accuracy of comparing endoscopists to the originating layer identification model in tertiary and binary classification

*P < 0.05, **P < 0.01, ***P < 0.001

### Interobserver agreement among endoscopists

In ternary categorization, interobserver agreement in distinguishing the originating layer was higher for senior endoscopists (*κ* = 0.430) than for junior endoscopists (*κ* = 0.227). In the binary categorization, the interobserver agreement between senior endoscopists distinguishing between the second or third layer with the fourth layer (*κ* = 0.419) was also higher than the interobserver agreement between junior endoscopists (*κ* = 0.257).

### Diagnostic time comparison of artificial intelligence models and endoscopists

When comparing the time required to complete the initial layer diagnosis, the junior endoscopists took an average of 6.78 s (ranging from 6.13 to 7.42 s) per image, whereas the senior endoscopists had an average of 4.03 s (ranging from 3.64 to 4.41 s). Conversely, the EUS-AI model required only 0.01 s, indicating that the model can achieve a diagnosis of more than 100 images per second.

## Discussion

EUS is an important method for identifying the location and originating layer of SELs [3]. However, the diagnostic accuracy predominantly depends on the expertise and experience of the endo-sonographers. CEUS encounters certain problems, such as unbalanced technical development, subpar diagnosis and treatment quality, and limited training facilities [16, 17, 29]. Therefore, in regions with a low EUS prevalence, the extensive development, diagnosis, and treatment of SEL diseases are limited. However, AI has the potential to address the demand for EUS by improving the diagnostic performance of junior endoscopists [30].

Previous studies have established AI models for the diagnoses of SELs [31, 32]. Hirai et al. [26] constructed a CNN model that classified five categories of SELs with an accuracy of 86.1%. For non-gastric SELs, Minoda et al. [33] developed an AI system to differentiate GISTs from non-GISTs, with an accuracy of 94.4%. However, these studies focused solely on the nature of the tumors. For classifying the origin of mural layers in the upper gastrointestinal tract, Li et al. [34] utilized data from 313 patients and developed an AI system with an 80.68% accuracy. However, the number of patients included in the study was limited.

Unlike previous studies, in this study, we developed an AI model using the YOLOv8s-seg algorithm for detecting esophageal SELs, used for analyzing esophageal SELs and constructed an AI model with the MobileNetv2 algorithm to differentiate the originating layer of the lesions, used for analyzing the accuracy. This represents a novel study in which AI was applied for lesion detection and layer identification in esophageal SELs, incorporating a substantial number of cases. This study demonstrates that the AI-based EUS diagnostic method exhibits high accuracy in identifying esophageal SELs, which is crucial for enhancing early diagnostic capabilities. With the assistance of the AI model, clinicians can more accurately assess the characteristics of lesions, thereby providing preliminary data support for the implementation of the AI model in real clinical settings. In the future, we anticipate that the model will be validated in larger and more diverse clinical samples to ensure its universality and effectiveness across various medical environments.

The AI model exhibited the highest AUC (0.832) and excellent diagnostic performance among the lesion models. Misdiagnosis of esophageal SELs may lead to the omission of malignant lesions and unnecessary treatment or follow-up. Consequently, using accuracy as the primary evaluation metric, we compared the diagnostic capabilities of the AI model with the endoscopists. The results indicated that the diagnostic performance of the AI model was comparable to that of senior endoscopists but significantly surpassed that of their junior counterparts. Hence, our AI model could be valuable for novice endoscopists in detecting esophageal SELs during EUS examinations.

The accuracy achieved by the endoscopists and AI system in the original layer of the recognition model was not as high as anticipated. In this study, only static images of the esophageal SELs were used. However, during endoscopy, endoscopists can enhance their view of the location of worrisome lesions by modifying or moving the sweep area. This aids in gathering additional information, ultimately leading to a more precise clinical diagnosis. Therefore, the test settings used in this study differ from the actual conditions encountered during endoscopy. Depending on the originating layer, size, and location of the esophageal SELs, appropriate treatment can be selected [3]. The accuracy of the EUS-AI model improved to 76.5% after the originating layers (second or third and fourth layers) were further binary-categorized, which was on par with the senior endoscopists but higher than that of the junior endoscopists. These results demonstrate the reliable diagnostic capability of the EUS-AI model.

Due to the subjective nature of the endoscopists’ diagnosis of EUS images, a lack of high interobserver agreement was revealed in this study, which emphasizes the necessity of standardized diagnostic tools. The measure of experience performing EUS is particularly important for diagnostic accuracy. Our AI model may effectively enhance the diagnostic accuracy of less-experienced endoscopists and reduce their learning duration. In addition, compared with endoscopists, the AI model has an extremely fast diagnostic speed (10 ms per image). It can provide rapid diagnosis using EUS, therefore significantly reducing diagnostic time and relieving the burden on endoscopists. In conclusion, the AI model is expected to function as a “secondary observer” during EUS examinations, serving as a backup resource to fully assist endoscopists in diagnosing and managing esophageal SELs.

There were some limitations in this study. First, this was a single-center retrospective study conducted at Shengjing Hospital, which excluded lower-quality EUS images and, therefore, may have been subject to selection bias. The diagnostic accuracy was not satisfactory, highlighting the necessity for future multicenter prospective studies to broaden the database for AI model training. Second, the limited inclusion of pathologically confirmed SELs in this study stems from the following reasons: (1) several esophageal SELs, such as cysts and lipomas, exhibit characteristic EUS features (e.g., anechoic cystic structures, homogeneous hyperechoic patterns) that enable reliable non-pathological diagnosis through follow-up observations; (2) a significant number of patients opt for surveillance over intervention; and (3) our endoscopic team (performing > 3000 annual EUS examinations) possess extensive experience and ensure diagnostic reliability. Although potential bias exists, we maintain that the direct utilization of EUS diagnostic outcomes does not undermine the credibility of model identification. Third, only image recognition of esophageal SELs was analyzed in this study, whereas video recognition and real-time experiments were not. The AI model accurately diagnosed EUS images at a rate of 10 ms/image. This is adequate for analyzing video images, indicating that real-time analysis will soon be achievable. Finally, one of the challenges with AI is the “black box problem.” This implies that only the input and output layers are visible, whereas the processes and recognition in the hidden layers remain unknown.

In conclusion, the EUS-AI model has high diagnostic performance for detecting lesions and identifying the originating layer in esophageal SELs. This model has the potential to enhance the proficiency of amateur endoscopists in diagnosing esophageal SELs during clinical practice, optimize diagnostic efficiency, and mitigate the impact of subjective bias on diagnostic accuracy. In the future, it will be essential to conduct prospective multicenter studies to develop a more precise and thorough AI system that may further enhance diagnostic performance.

## Supplementary Information

Below is the link to the electronic supplementary material.

Supplementary file1 (DOCX 17 KB)

Supplementary file2 (DOCX 19 KB)

Supplementary file3 (DOCX 18 KB)

## Data Availability

The model source code can be accessed through this link: https://github.com/GXDD666/SMT-E.
