# Supplementary material for: Artificial intelligence-assisted endoscopic ultrasound diagnosis of esophageal subepithelial lesions
Source: Surg Endosc. 2025 May 7;39(6):3821–31. doi: 10.1007/s00464-025-11767-5 (PMC12116721; doi:10.1007/s00464-025-11767-5)
Supplement: Supplementary file 1 — Supplementary file1 (DOCX 17 KB) [file 464_2025_11767_MOESM1_ESM.docx]

Supplementary Table 1 Performance of the AI models in lesion detection

|  | Precision | Recall |
| --- | --- | --- |
| Yolov8s-seg | 92.2% | 73.6% |
| Yolov8 | 91.6% | 72.9% |
| Yolov5s | 91.4% | 72.7% |

AI, artificial intelligence.
