# Supplementary material for: Artificial intelligence-assisted endoscopic ultrasound diagnosis of esophageal subepithelial lesions
Source: Surg Endosc. 2025 May 7;39(6):3821–31. doi: 10.1007/s00464-025-11767-5 (PMC12116721; doi:10.1007/s00464-025-11767-5)
Supplement: Supplementary file 2 — Supplementary file2 (DOCX 19 KB) [file 464_2025_11767_MOESM2_ESM.docx]

Supplementary Table 2 Accuracies of the AI models in originating layer recognition

|  | The second layer | The third layer | The fourth layer | All |
| --- | --- | --- | --- | --- |
| MobileNetv2 | 50.0% | 54.5% | 60.0% | 55.2% |
| Resnet50 | 47.4% | 52.6% | 58.0% | 53.0% |
| EfficientnetB0 | 46.6% | 50.9% | 57.1% | 51.9% |

AI, artificial intelligence.
