# Supplementary material for: Artificial intelligence-assisted endoscopic ultrasound diagnosis of esophageal subepithelial lesions
Source: Surg Endosc. 2025 May 7;39(6):3821–31. doi: 10.1007/s00464-025-11767-5 (PMC12116721; doi:10.1007/s00464-025-11767-5)
Supplement: Supplementary file 3 — Supplementary file3 (DOCX 18 KB) [file 464_2025_11767_MOESM3_ESM.docx]

Supplementary Table 3 Hyperparameter settings

| Hyperparameter | YOLOv8s-seg | MobileNetv2 |
| --- | --- | --- |
| Input size (pixels) | 512 × 512 | 320 × 320 |
| Batch size | 32 | 32 |
| Epoch | 100 | 70 |
| Optimizer | Stochastic gradient descent | Adaptive moment estimation |
| Initial learning rate | 0.01 | 0.001 |
| Final learning rate | 0.0001 | 0.000001 |
| Momentum | 0.937 | [0.9, 0.999] |
